# Predictors of antibiotic prescription for upper respiratory infections in cancer patients

**DOI:** 10.1017/ash.2025.10180

**Published:** 2025-10-27

**Authors:** Hyeon Mu Jang, Aida Boumiza, I-Hsin Lin, Krishna Shah, Melvili Cintron, Jibran Majeed, Jeffrey Groeger, Nina Cohen, Susan K. Seo

**Affiliations:** 1 Infectious Disease Service, Department of Medicine, Memorial Sloan Kettering Cancer Center, New York, NY, USA; 2 Biostatistics Service, Department of Epidemiology and Biostatistics, Memorial Sloan Kettering Cancer Center, New York, NY, USA; 3 Department of Pharmacy, Memorial Sloan Kettering Cancer Center, New York, NY, USA; 4 Department of Pathology and Laboratory Medicine, Memorial Sloan Kettering Cancer Center, New York, NY, USA; 5 Advanced Practice and Patient Care Service, Memorial Sloan Kettering Cancer Center, New York, NY, USA; 6 Emergency Care Service, Department of Medicine, Memorial Sloan Kettering Cancer Center, New York, USA; 7 Department of Medicine, Weill Cornell Medical College, New York, NY, USA

## Abstract

**Background:**

Limited data regarding predictors of antibiotic prescription for acute upper respiratory infections (URI) are available for ambulatory patients with cancer.

**Methods:**

Adult cancer patients with URI who presented to the Urgent Care Center or Symptom Care Clinic (SCC) and had a respiratory pathogen panel (RPP) between September 1, 2019 and March 11, 2020, were evaluated. Patients were dichotomized by receipt of antibiotic prescription (yes vs no). Demographics, cancer treatment, URI characteristics, and URI management were compared by Student’s t test or Mann–Whitney U test and χ^2^ or Fisher’s exact test as appropriate. Logistic regression was performed to evaluate predictors of antibiotic prescription.

**Results:**

Of 552 patients, 384 (69.6%) received cancer treatment within 30 days of the URI, and 377 (68.3%) had a positive RPP result. Antibiotics were prescribed in 156 (28.3%) patients, and 26/156 (16.7%) were appropriate. Multivariate logistic regression showed that predictors of antibiotic prescribing included SCC visit location (odds ratio (OR) 2.09, 95% confidence interval (CI) 1.30–3.38), symptom duration >7 days (OR 2.29, 95% CI 1.26 – 4.17), earache (OR 9.30, 95% CI 3.47 – 24.93), sinus symptoms (OR 2.64, 95% CI 1.53 – 4.56), negative RPP result (OR 1.88, 95% CI 1.18 – 2.98), and negative RPP result for influenza (OR 3.31, 95% CI 1.53 – 7.16).

**Conclusions:**

Almost one-third of outpatients at a single cancer center were prescribed antibiotics for URI with only 16.7% being appropriate. Getting real-time RPP results can be helpful to optimize antibiotic prescribing. Understanding risk factors for antibiotic prescribing in ambulatory cancer patients with URI may better direct antimicrobial stewardship efforts.

## Introduction

Acute upper respiratory infections (URIs), which include acute uncomplicated bronchitis, pharyngitis, rhinosinusitis, and the common cold, are the most common reason for ambulatory visits.^1^ In the United States, these conditions have collectively been associated with 221 antibiotic prescriptions per 1000 patients, of which 50% is deemed inappropriate.^2^ The Centers for Disease Control and Prevention (CDC) identifies acute URIs as examples of high-priority conditions in which clinicians commonly deviate from best practices for antibiotic prescribing and for which there are opportunities to improve outpatient antibiotic stewardship.^1,3^ Factors that influence antibiotic prescribing for acute URIs, including those that are institution-specific, would be important for antimicrobial stewardship programs to recognize as they think about quality improvement initiatives in this area.

Multiple studies on acute URIs in ambulatory settings have examined factors associated with antibiotic prescribing in the general patient population.^4–13^ Healthcare provider characteristics such as type, number of years since training graduation, and specialty appear to drive some of the variations in antibiotic prescription.^4,6,9,12^ Others have found that certain diagnoses (eg, acute sinusitis) or clinical features (eg, purulent sputum, tonsillar exudate) can influence antibiotic prescribing.^6–8^ Additionally, rapid diagnostics to detect respiratory viruses, particularly influenza, may impact treatment decisions.^11^

In contrast, not much is known about how ambulatory cancer patients with acute URIs are managed. Community-acquired respiratory viral infections in immunosuppressed cancer patients may lead to pneumonia in approximately 30% of cases, so rapid diagnosis is important to guide decisions regarding infection control, antiviral therapy if indicated, and timing of cancer treatment.^14^ If there is diagnostic uncertainty regarding the causality of a cancer patient’s URI symptoms, it is plausible that this may affect a provider’s decision to prescribe an antibiotic.

At one cancer center, nearly one-third of hematology-oncology patients were given antibiotic prescriptions for URIs.^15^ Patients who presented with sputum production or chest congestion were more likely to have received an antibiotic. Another finding was that testing for respiratory viruses was associated with a lower risk for antibiotic prescribing. However, respiratory viral testing was predominantly performed by the bone marrow transplant and hematology services and was rarely ordered in the solid tumor clinics. The high correlation between respiratory viral testing and clinical service thus limited the authors’ ability to separate these effects on prescribing. It was also not clear whether these results were unique to their institution.

At Memorial Sloan Kettering Cancer Center (MSK), respiratory pathogen panel (RPP) testing is readily available at all outpatient locations. Adult cancer patients who presented to the hospital’s Urgent Care Center (UCC) or Symptom Care Clinic (SCC) with URI symptoms and underwent RPP testing were examined. Study objectives were to characterize URI management and to determine predictors of antibiotic prescription.

## Methods

### Study population

This study was reviewed and approved by the Institutional Review Board at MSK, a tertiary cancer center in New York, NY. Patients with urgent medical issues related to cancer or their treatment can be seen in the UCC at the hospital’s main campus or at a SCC located at one of six regional sites within New York and New Jersey. Adults ≥ 18 years of age who presented to the hospital’s UCC or SCC with URI symptoms and underwent RPP testing between September 1, 2019, and March 11, 2020, were included. For patients who had > 1URI episode, only the first episode was included in the analysis.

Any patient with URI symptoms presenting to the UCC or SCC after March 11, 2020, was excluded due to the start of the global Coronavirus Disease 2019 (COVID-19) pandemic.^16^ Patients were also excluded if they had RPP testing but no documented URI symptoms, if they had evidence for lower respiratory tract involvement,^17^ or if they required 24-hour observation or hospital admission immediately after the UCC or SCC encounter.

The institutional electronic health record was used to obtain clinical information, as well as laboratory, microbiology, and radiology results, for each patient encounter. Abstracted data encompassed four broad areas: (1) patient attributes (eg, demographics, cancer diagnosis, comorbid conditions); (2) cancer treatment; (3) visit (eg, date, location, provider type) and URI characteristics; and (4) URI management.

### Definitions

Neutropenia was defined as having an absolute neutrophil count<1.0 K/mcL; lymphopenia was defined as having an absolute lymphocyte count < 0.9 K/mcL. The appropriateness of the antibiotic prescription for indication was determined by using CDC guidance for adults seeking care for common infections in the outpatient setting.^18^ Documentation pertaining to counseling on supportive care measures (eg, ensure adequate hydration, use decongestant as needed) and/or return instructions if patients had worsening symptoms counted as patient education. Clinic or telephone follow-up within 14 days of the initial encounter was defined as URI-related if any of the following was conducted: notification of the RPP result, URI symptom follow-up, counseling on supportive care measures for the URI, antibiotic or antiviral management, resumption in cancer treatment due to recovery from the URI, or delay in cancer treatment due to ongoing symptoms.

### Laboratory methods

Nasopharyngeal swabs were submitted for respiratory pathogen testing at the clinicians’ discretion. Testing was performed by using the FilmArray® Respiratory Panel that could detect adenovirus; coronavirus types 229E, HKU1, NL63, and OC43; human metapneumovirus; human rhinovirus/enterovirus; influenza types A and B; parainfluenza types 1, 2, 3, and 4; respiratory syncytial virus; *Bordetella parapertussis*, *B. pertussis*, *Chlamydophila pneumoniae*, and *Mycoplasma pneumoniae* (BioFire Diagnostics Inc., Salt Lake City, UT, USA).^19^

### Statistical analyses

Descriptive statistics were presented as counts and percentages for categorical variables and as means and standard deviations (SD) or medians and interquartile ranges (IQR) for continuous variables. To compare baseline characteristics between patients who received antibiotic prescriptions and those who did not, the χ^2^ or Fisher’s exact test was performed for categorical variables, and the Student’s t test or Mann–Whitney U test was performed for continuous variables. Univariate and multivariate logistic regression analyses were used to evaluate predictors of antibiotic prescription for URIs in cancer patients. SAS software version 9.4 (SAS Institute Inc., Cary, NC, USA) was used in all analyses. All statistical tests were 2-tailed, and *P*-values < .05 were considered statistically significant.

## Results

### Patient characteristics

A total of 552 patients comprised the study population. Overall, the mean age (SD) was 57.8 (14) years, 216 (39.1%) were men, and the majority (343, 62.1%) had solid tumor cancers. Only 101 (18.3%) individuals were recipients of hematopoietic cell transplantation (HCT) or chimeric antigen receptor T-cell therapy. Study patients had a median (IQR) of one (0 – 2) comorbid condition besides their cancer, and many (384, 69.6%) underwent cancer therapy within 30 days of the UCC or SCC visit.

Table 1 shows patient characteristics dichotomized by antibiotic prescription status. One hundred fifty-six patients (28.3%) were prescribed an antibiotic for their URI at the UCC or SCC visit. A significantly higher proportion of patients who got an antibiotic had receipt of cancer therapy within 30 days of the visit compared with those who did not receive an antibiotic (120/156, 76.9% vs 264/396, 66.7%; *P* = .018).

**Table 1.** Patient characteristics by antibiotic prescription status

	Antibiotic prescription			
	No (*N* = 396)	Yes (*N* = 156)	Total (*N* = 552)	*P* -value
**Age (years), mean (SD)**	57.5 (14.4)	58.5 (12.9)	57.8 (14)	.45
**Male, *N* (%)**	165 (41.7)	51 (32.7)	216 (39.1)	.05
**Race, *N* (%)**				
Black	044 (11.5)	011 (7.1)	055 (10.2)	.08
White	288 (75)	130 (83.9)	418 (77.6)	
Asian/Other	052 (13.5)	014 (9)	066 (12.2)	
**Cancer diagnosis, *N* (%)**				
Hematologic	150 (37.9)	059 (37.8)	209 (37.9)	.99
Solid	246 (62.1)	097 (62.2)	343 (62.1)	
**HCT or cellular therapy, *N* (%)**				
Allogeneic	032 (43.2)	009 (33.3)	041 (40.6)	.05
Autologous	042 (56.8)	016 (59.3)	058 (57.4)	
CART-cell therapy	000 (0)	002 (7.4)	002 (2)	
**ANC, median (IQR)**	3.8 (2.3 – 5.6)	4.4 (2.3 – 6.7)	3.9 (2.3 – 5.8)	.18
**ALC, median (IQR)**	.9 (.5 – 1.4)	1.0 (.6 – 1.2)	.9 (.5 – 1.3)	.80
**Lymphopenia, *N* (%)**	184 (47.3)	066 (43.1)	250 (46.1)	.38
**Cancer therapy within 30 days, *N* (%)**	264 (66.7)	120 (76.9)	384 (69.6)	.018*
**GCSF receipt, *N* (%)**	060 (15.2)	029 (18.6)	089 (16.1)	.32
**Number of comorbidities, median (IQR)**	1.0 (.5 – 2.0)	1.0 (.0 – 2.0)	1.0 (.0 – 2.0)	.70
**Comorbid condition, *N* (%)**				
HIV	002 (.5)	000 (0)	002 (.4)	--
Gastrointestinal	112 (28.3)	040 (25.6)	152 (27.5)	.53
Pulmonary	063 (15.9)	031 (19.9)	094 (17)	.26
Renal	021 (5.3)	005 (3.2)	026 (4.7)	.29
Cardiovascular	174 (43.9)	078 (50)	252 (45.7)	.20
Dementia	001 (.3)	001 (.6)	002 (.4)	.49
Endocrine	109 (27.5)	046 (29.5)	155 (28.1)	.64
Rheumatologic	023 (5.8)	011 (7.1)	034 (6.2)	.58
Neurologic	047 (11.9)	017 (10.9)	064 (11.6)	.75

Abbreviations: ALC, absolute lymphocyte count; ANC, absolute neutrophil count; CAR, chimeric antigen receptor; GCSF, granulocyte colony stimulating factor; HCT, hematopoietic cell transplantation; HIV, human immunodeficiency virus; IQR, interquartile range; SD, standard deviation.

### Visit and URI characteristics

**Figure 1.**
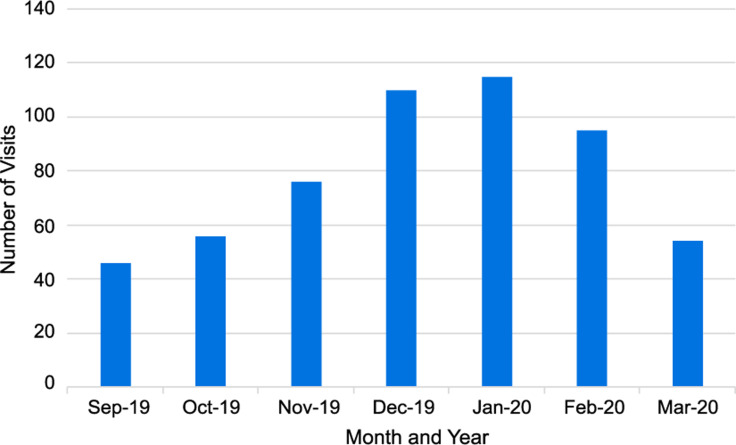
Distribution of visits for upper respiratory infection by month.

**Figure 2.**
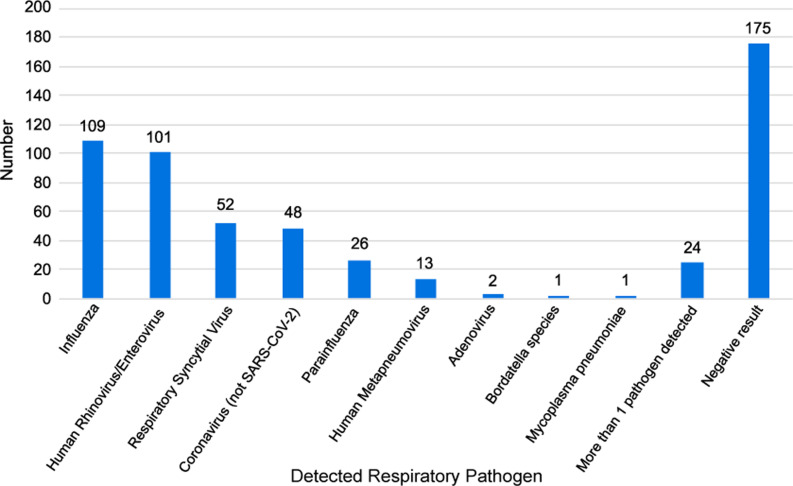
Respiratory pathogen panel results.

Study patients were seen by a physician (252, 45.7%) or an advanced practice provider (300, 54.3%), respectively. Many (302, 54.7%) were evaluated at the SCC. The distribution of visits for URI by month is shown in Figure 1. The majority (439, 86.6%) presented with symptom duration ≤ 7 days with a median (IQR) of 3 (2 – 3) reported symptoms. Of 552 RPP tests, 377 (68.3%) were positive, and 122 (22.1%) were positive for influenza type A or B. Figure 2 shows the distribution of RPP results. In addition to the RPP test, 9 (1.6%) had sputum sent for culture, and 92 (16.7%) underwent throat culture. In the univariate analysis, there were statistical differences in visit location, positive RPP result, positive RPP result for influenza, symptom duration, earache, sinus symptoms, fever, and pharyngitis between cancer patients who received an antibiotic for their URI and those who did not (Table 2).

**Table 2.** Visit and URI characteristics by antibiotic prescription status

	Antibiotic Prescription			
	No (*N* = 396)	Yes (*N* = 156)	Total (*N* = 552)	*P* Value
**Provider, *N* (%)**				
Physician	185 (46.7)	67 (42.9)	252 (45.7)	.42
APP	211 (53.3)	89 (57.1)	300 (54.3)	
**Visit location, *N* (%)**				
Symptom Care Clinic	188 (47.5)	114 (73.1)	302 (54.7)	< .001
Urgent Care Center	208 (52.5)	42 (26.9)	250 (45.3)	
**Positive RPP result, *N* (%)**	295 (74.5)	82 (52.6)	377 (68.3)	< .001
**RPP positive for influenza, *N* (%)**	112 (28.3)	10 (6.4)	122 (22.1)	< .001
**Symptom duration, *N* (%)**				
Within 1 week	324 (89.5)	115 (79.3))	439 (86.6)	< .005
More than 1 week	38 (10.5)	030 (20.7)	68 (13.4)	
**Number of symptoms, median (IQR)**	2.0 (2.0 – 3.0)	3.0 (2.0 – 3.0)	3.0 (2.0 – 3.0)	.09
**URI symptoms, *N* (%)**				
Nasal congestion	206 (52)	82 (52.6)	288 (52.2))	.82
Earache	6 (1.5)	22 (14.1)	28 (5.1)	< .001
Myalgia	55 (13.9)	19 (12.2)	074 (13.4)	.60
Sinus symptoms	48 (12.1)	45 (28.8)	93 (16.8)	< .001
Fever	200 (50.5)	54 (34.6)	254 (46)	< .001
Cough	308 (77.8)	119 (76.3)	427 (77.4)	.71
Shortness of breath	33 (8.3)	10 (6.4)	43 (7.8)	.45
Cervical lymphadenopathy	2 (.5)	1 (.6)	3 (.5)	1.0
Pharyngitis	109 (27.5)	57 (36.5)	166 (30.1)	< .05
Other symptoms	39 (9.8)	15 (9.6)	54 (9.8)	.93

Abbreviations: APP, advanced practice provider; IQR, interquartile range; RPP, respiratory pathogen panel; URI, upper respiratory infection.

### URI management

The top three prescribed antibiotics were azithromycin, amoxicillin/clavulanate, and levofloxacin, accounting for 86.5% (135/156) of prescriptions (Table 3). Only 26 of 156 (16.7%) prescriptions had appropriate indications for antibiotics and included 2 with group A streptococcal pharyngitis; 6 with either acute bronchitis, otitis media, or sinusitis with concomitant neutropenia; 5 with acute bronchitis but with concern for progression to pneumonia if untreated; 9 with acute otitis media; and 4 with persistent or worsening symptoms of sinusitis. Three of 156 (1.9%) patients subsequently reported an adverse reaction to the antibiotic and discontinued. Providers’ suggestions for symptom relief such as use of decongestants or cough suppressants are shown in Table 3. Overall, 323 (58.5%) patients received counseling on supportive care measures, and 288 (52.2%) patients were advised to return if they had worsening symptoms. Among patients who were actively receiving treatment for their malignancy, 19% (73/384) had cancer treatment held due to the URI diagnosis.

**Table 3.** Upper respiratory infection management by antibiotic prescription status

	Antibiotic Prescription		
	No (*N* = 396)	Yes (*N* = 156)	Total (*N* = 552)
**Antibiotic prescription appropriateness, *N* (%)**	--	26 (16.7)	--
**Prescribed antibiotic, *N* (%)**			
Amoxicillin	--	003 (1.9)	--
Amoxicillin/Clavulanate	--	038 (24.4)	--
Azithromycin	--	078 (50)	--
Cefpodoxime	--	001 (.6)	--
Cefuroxime + Azithromycin	--	001 (.6)	--
Cephalexin	--	003 (1.9)	--
Doxycycline	--	011 (7.1)	--
Levofloxacin	--	019 (12.2)	--
Penicillin	--	002 (1.3)	--
**Other treatment measures, *N* (%)**			
Nasal irrigation	006 (1.5)	004 (2.6)	010 (1.8)
Decongestant	042 (10.6)	020 (12.8)	062 (11.2)
Antiviral (oseltamivir)	95 (24)	2 (1.3)	97 (17.6)
Antitussive	083 (21.0)	034 (21.8)	117 (21.2)
Antipyretic	52 (13.1)	13(8.3)	65 (11.8)
Nasal steroid	018 (4.5)	008 (5.1)	026 (4.7)
Nasal spray	008 (2.0)	003 (1.9)	011 (2.0)
Oral steroid	003 (.8)	001 (.6)	004 (.7)
Antihistamine	019 (4.8)	008 (5.1)	027 (4.9)
Inhaler	029 (7.3)	012 (7.7)	041 (7.4)
Warm rinses or lozenges	016 (4.0)	004 (2.6)	020 (3.6)
**Type of patient education provided, *N* (%)**			
Supportive care measures	242 (61.1)	081 (51.9)	323 (58.5)
Return precautions	207 (52.3)	081 (51.9)	288 (52.2)
**14-day follow-up, *N* (%)**			
In-person	082 (30.3)	046 (41.4)	128 (33.5)
Telephone	189 (69.7)	065 (58.6)	254 (66.5)

Many study patients (382/552, 69.2%) had telephone or in-person follow-up with their primary oncology team within 14 days of the initial encounter. Details of URI-related interventions during follow-up are shown in Table 4. Among 382 patients who had follow-up, 4 (1%) patients were instructed to stop the antibiotic based on the RPP result supporting a viral etiology, and 26 (6.9%) were prescribed oseltamivir for a positive RPP result for influenza. Ten of 382 (2.6%) patients were directed for admission due to pneumonia (*N* = 7), dehydration in setting of influenza (*N* = 2), and fever and neutropenia (*N* = 1).

**Table 4.** URI-related interventions performed during the follow-up encounter

	Total (*N* = 382)
Notification of RRP result, *N* (%)	93 (24.3)
URI symptom follow-up, *N* (%)	261 (68.3)
Counseling on supportive care measures, *N* (%)	95 (24.9)
Antibiotic or antiviral management, *N* (%)*	30 (7.9)
Resumption of cancer treatment, *N* (%)	60 (15.7)
Delay in cancer treatment, *N* (%)	39 (10.2)
Admission, *N* (%)	10 (2.6)

Abbreviations: RPP, respiratory pathogen panel; URI, upper respiratory infection

Note. Patients could have more than one URI-related intervention during the follow-up encounter.

* Based on the RPP result, 26 patients were prescribed oseltamivir for influenza, and 4 patients were instructed to stop taking the antibiotic.

### Predictors of antibiotic prescribing

SCC visit location, cancer therapy within 30 days of the UCC or SCC visit, symptom duration > 7 days, earache, sinus symptoms, pharyngitis, negative RPP result, and negative RPP result for influenza correlated with likelihood of antibiotic prescription by univariate logistic regression (Table 5). Multivariate logistic regression showed that predictors of antibiotic prescribing remained SCC visit location (odds ratio (OR) 2.09, 95% confidence interval (CI) 1.30 – 3.38), symptom duration > 7 days (OR 2.29, 95% CI 1.26 – 4.17), earache (OR 9.30, 95% CI 3.47 – 24.93), sinus symptoms (OR 2.64, 95% CI 1.53 – 4.56), negative RPP result (OR 1.88, 95% CI 1.18 – 2.98), and negative RPP result for influenza (OR 3.31, 95% CI 1.53 – 7.16) (Table 5).

**Table 5.** Univariate and multivariate models for predictors of antibiotic prescription

	Univariate ModelOR (95% CI)	*P* value	Multivariate ModelOR (95% CI)	*P*-value
Visit location (UCC vs SCC)	3.00 (2.00 – 4.50)	< .0001	2.09 (1.30 – 3.38)	< .005
Cancer therapy within 30 days (No vs Yes)	1.67 (1.09 – 2.55)	.02	1.25 (.75 – 2.08)	.40
Provider (Physician vs APP)	1.17 (0.80 – 1.69)	.42	--	--
Symptom duration (< = 7 d vs > 7 d)	2.23 (1.32 – 3.76)	< .0001	2.29 (1.26 – 4.17)	< .01
Earache (No vs Yes)	10.67 (4.24 – 26.88)	< .0001	9.30 (3.47 – 24.93)	< .0001
Sinus symptoms (No vs Yes)	2.94 (1.86 – 4.65)	< .0001	2.64 (1.53 – 4.56)	< .0001
Fever (No vs Yes)	0.52 (0.35 – 0.76)	< .001	0.96 (0.59 – 1.56)	.87
Pharyngitis (No vs Yes)	1.52 (1.02 – 2.25)	.04	1.23 (0.76 – 1.97)	.40
RPP result (Positive vs Negative)	2.64 (1.79 – 3.88)	< .0001	1.88 (1.18 – 2.98)	< .01
Diagnosis of influenza (Yes vs No)	5.76 (2.93 – 11.33)	< .0001	3.31 (1.53 – 7.16)	< .005

Note. The categories on the left side of vs are the reference groups. The categories on the right side of vs are the comparison groups of interest.Abbreviations: APP, Advanced Practice Provider; CI, confidence interval; OR, odds ratio; RPP, respiratory pathogen panel; SCC, Symptom Care Clinic; UCC, Urgent Care Center.

## Discussion

In this study of 552 ambulatory cancer patients who underwent RPP testing for their acute URI, an antibiotic was prescribed in 156 (28.3%) patients. Seven (4.5%) of 156 patients were subsequently instructed to stop taking the antibiotic due to either an adverse reaction or confirmation of a viral etiology on the RPP test during follow-up with their primary oncology team. In addition, all patients diagnosed with influenza were prescribed oseltamivir with the majority (79.5%) of prescriptions provided at the UCC or SCC visit. The risk of antibiotic prescription was significantly increased with the SCC visit location, symptom duration > 7 days, symptom type (namely, earache and sinus symptoms), negative RPP result, and negative RPP result for influenza.

Cancer patients are vulnerable to infections through an interplay of underlying immune deficiencies, associated comorbidities, and treatment-related adverse effects.^20^ It has not been clear whether providers would be more inclined to give antibiotics for acute URIs in this special patient population. This study found that antibiotics were prescribed for URIs in 28.3% of hematology-oncology outpatients. Similarly, Krantz et al found that 32% of patients were given antibiotics in the only other publication consisting solely of ambulatory cancer patients with URIs.^15^ Krantz et al also found that antibiotic prescribing was significantly lower among those who got respiratory viral testing, but because respiratory viral testing was primarily done in bone marrow transplant recipients and patients with hematologic malignancies, they were unable to gauge how access to testing would have influenced prescribing in the solid tumor clinics. At MSK, RPP testing is available at all ambulatory sites, so patients with solid tumors or hematologic malignancies are well represented.

We found that RPP results were significant predictors for antibiotic prescribing in ambulatory cancer patients with URIs. Regardless of cancer type, patients had a 1.88-fold likelihood of being prescribed an antibiotic with a negative RPP result and a 3.31-fold likelihood of being prescribed an antibiotic with a negative RPP result for influenza. Previous investigations in immunocompetent outpatients have likewise suggested that multiplex real-time polymerase chain reaction assays for the detection of respiratory pathogens may decrease unnecessary antibiotic use.^21,22^

Even so, patients can still be prescribed antibiotics despite the finding of a respiratory virus. Of the 156 patients given an antibiotic in our study, 82 (52.6%) had a positive RVP result. Krantz et al also noted that antibiotics were prescribed despite viral etiologies being identified in 75% of those tested.^15^ Part of this is because detection of a virus by sensitive molecular methods reduces but does not eliminate the probability of a secondary bacterial infection.^23^ There are also lack of data on which cancer patient subgroups outside of allogeneic HCT recipients and patients with hematologic malignancies will progress to lower respiratory tract infection.^24^ It would be helpful to have validated scoring tools like the immunodeficiency scoring index that stratifies allogeneic HCT patients with respiratory syncytial virus to distinguish those who would benefit most from antiviral therapy.^25^ Finally, providers may discharge patients from their clinic while respiratory viral results are still pending. While the SCC visit location was a predictor of antibiotic prescription, we think that this finding was instead a surrogate marker for the longer transit times needed for transport of the nasopharyngeal swabs from the individual SCCs within the NY and NJ region to the clinical microbiology laboratory in comparison with the UCC that is in closer proximity. Thus, providers in the UCC received timelier results.

Certain reported symptoms corroborated by physical examination findings were associated with antibiotic receipt, but only earache and sinus symptoms remained statistically significant. There are no national treatment guidelines for acute otitis media (AOM) in adults. Some experts suggest that it may be prudent to treat since this entity is unusual in adults and complications (eg, mastoiditis, brain abscess) while rare can be significant.^26^ However, one large VA study found no difference in outcomes between patients who received antibiotics and those who did not, suggesting that watchful waiting may be an appropriate strategy for most.^27^ Because of sparse data in cancer patients, we chose to define antibiotic treatment for AOM as appropriate. Investigations to identify risk factors for complicated AOM in adults to better target those most likely to benefit from antibiotic treatment are needed.

National guidelines for acute rhinosinusitis recommend symptomatic therapy for all patients and an observation period for most of the patients with uncomplicated infection.^18^ Antibiotic treatment for acute bacterial sinusitis is contingent on severity, persistence (> 10 d) without improvement, or biphasic worsening of symptoms. Despite this framework, antibiotics continue to be commonly given before waiting at least 10 days for symptoms to subside.^6,11,28^ One problem with the guidelines is that there are too many variables to consider during the ambulatory visit. Perhaps scheduling follow-up for these patients rather than giving them a prescription may be a better approach.

Having symptom duration > 7 days was also found to be a predictor. To our knowledge, this finding has not been previously described in the published literature but doesn’t seem surprising. While the usual duration of acute URIs is 7 – 10 days, symptoms can linger for up to 21 days. This is of relevance to cancer patients who can experience delays in their cancer treatment pending symptom resolution. Nearly 20% of our study patients had cancer treatment held due to the URI diagnosis.

One unique aspect of our study was that almost 70% of patients had documentation of follow-up within 14 days of the initial UCC or SCC visit. This contrasts with the general patient population in which scheduling routine clinic follow-ups for an acute URI is not the norm. In our study, few patients required hospital admission, which is concordant with that of Krantz et al who found that serious outcomes related to acute URIs (eg, lower respiratory tract infections, hospitalizations) were rare.^15^ These data suggest that management of acute URIs for most cancer patients should not differ significantly from that of non-cancer patients.

When examining interventions performed by the primary oncology teams during telephone or scheduled clinic follow-up, the most common intervention was checking for symptom resolution, followed by counseling on supportive care measures (if URI symptoms were still present), informing the patient of the RPP result, and deciding whether to resume or delay cancer treatment. It is notable that 30 (7.9%) of the interventions entailed antibiotic or antiviral management in which 26 patients were prescribed oseltamivir and 4 were instructed to stop taking the antibiotic. From an antimicrobial stewardship perspective, the telephone or in-person follow-up visit may be an opportunity to educate and develop a protocol to assist primary oncology teams on when to stop unnecessary antibiotics for viral URIs.

In terms of study limitations, results may not be generalizable to other cancer centers that may have differing patient populations, ambulatory practices, and respiratory viral testing procedures. Definitions for what constituted appropriate antibiotic therapy (eg, treatment of acute AOM) may also differ. However, we also have several strengths. One is that we had a large study cohort with good representation of adults with solid tumor or hematologic malignancies with acute URIs, and all underwent RPP testing as part of their workup. This enabled us to characterize their URI presentation and management. In addition, we were able to follow their URI course due to the majority having telephone or clinic follow-up within 14 days of the initial visit. This finding is something that we can try to leverage when we think about antimicrobial stewardship initiatives to reduce needless antibiotic use for acute URI indications.

In conclusion, almost one-third of outpatients at a single cancer center were prescribed antibiotics for their acute URI with only 16.7% being appropriate. Having a negative RPP result or a negative RPP result for influenza were two predictors of antibiotic prescription. Other predictors included prolonged URI symptom duration, earache, and sinus symptoms. Understanding these risk factors for antibiotic prescribing in ambulatory cancer patients with URIs may better direct antimicrobial stewardship efforts.
